# MicroRNA (miRNA) in cancer

**DOI:** 10.1186/s12935-015-0185-1

**Published:** 2015-04-02

**Authors:** Kaladhar B Reddy

**Affiliations:** Department of Pathology, Wayne State University School of Medicine, 540 E. Canfield Anvenue, Detroit, MI 48201 USA; Karmanos Cancer Institute, Wayne State University, Detroit, MI USA

**Keywords:** miRNA, Cancer, DNA methylation, Single nucleotide polymorphism, Diagnosis, Therapy

## Abstract

In recent years, there has been a tremendous and growing interest among researchers to investigate the role of mircoRNA (miRNA) in normal cellular as well as in disease processes. miRNAs are a family of small non-coding RNAs which were reported to regulate the expression of various oncogenes or tumor suppressor genes. The expression profiling of miRNAs has already entered into cancer clinics as diagnostic and prognostic biomarkers to assess tumor initiation, progression and response to treatment in cancer patients. This review summarizes: (i) the current understanding of interactions between miRNAs and their target genes, (ii) recent advances in the regulatory mechanisms that control the expression of genes related to carcinogenesis, and (iii) the role of miRNAs in cancer diagnosis and therapy.

## Introduction

MicroRNAs (miRNAs) are small non-coding regions in RNAs of 20–22 nucleotides, which play an important role in all biological pathways in multicellular organisms including mammals [1]. Under normal physiological conditions, miRNAs function in feedback mechanisms by safeguarding key biological processes including cell proliferation, differentiation and apoptosis [2,3]. De-regulation of a single or small subset of miRNAs was reported to have a profound effect on the expression pattern of several hundred mRNAs [4,5] which propels the cells towards transformation [6,7]. The human disease-related miRNAs, viz., miR15 and miR16 at 13q14, were first characterized in chronic lymphocytic leukemia [8,9]. Subsequently, elevated levels of tumor-associated miRNAs were identified in the serum of patients with diffuse large B-cell lymphoma [8,9]. Emerging evidence has also suggested the involvement of long noncoding RNA (IncRNA) in the development and progression of cancer [10] by exerting their regulatory functions through specific interactions with proteins, including epigenetic modifiers, transcriptional factors/co-activators, and RNP complex [11-13]. In this review, the role of miRNAs in carcinogenesis/cancer is discussed.

### Biosynthesis of miRNA

**Figure 1:**
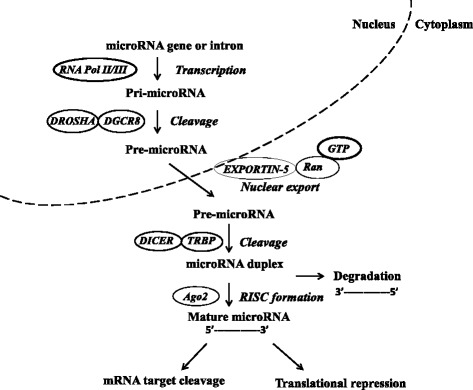
**MicroRNA biogenesis pathways and their regulation: a schematic representation depicting the miRNA biogenesis pathway.** The primary miRNA transcript (pri-miRNA) by RNA polymerase II or III and cleavage of the pri-miRNA by the microprocessor complex Drosha-DGC8 in the nucleus. The resulting precursor hairpin, the pre-miRNA, is exported from the nucleus by exportin-5-Ran-GTP. In the cytoplasm, the RNase Dicer form complex with the double-stranded RNA-binding protein TRBP cleaves the pre-miRNA hairpin to its mature length. The functional strand of the mature miRNA is loaded together with Argonaute (Ago2) proteins into the RNA-induced silencing complex (RISC), where it targets mRNAs through mRNA cleavage, deadenylation or translational repression, where as passenger strand (3′---------5′) is degraded.

The biogenesis of miRNA is schematically presented in Figure 1. Generally, it involves transcription of a pri-miRNA precursor by RNA polymerase II which is subsequently processed in the nucleus by endonuclease enzymes such as DROSHA and DGCR8 resulting in pre-miRNA sequence consisting of approximately 80–100 nucleotides [14,15]. Exportin-5 was reported to assist in the transport of pre-miRNAs from the nucleus to the cytoplasm [16] where a cytoplasmic ribonuclease, Dicer, cleaves it into double stranded mature miRNA [17]. Then, the mature miRNA duplex binds to Argonaute (Ago) proteins forming RNA-induced silencing complex (RISC) which then regulates the translation of complementary messenger RNA (mRNA). The mature miRNA recognizes its complementary sequences in the 3′ untranslated region (UTR) of their target mRNAs via seed region, typically positions 2–7 in the miRNA. Recent studies have suggested that miRNAs binds to 5′UTR or open reading frame (ORF) of the target mRNA [18,19]. Since high complementarity is not required for regulation, a single miRNA may target up to several hundred mRNAs and the resulting aberrant miRNA expression may affect a multitude of transcripts, which have profound influence on cancer-related signaling pathways.

### Involvement of microRNAs in cancer

There were several reports indicating more than half of the miRNAs genes are located in cancer-associated genomic regions or in fragile sites. Microarray expression data from a wide spectrum of cancer tissues/cells have shown that aberrant miRNA expression is a rule rather than exception. The involvement and the role played by miRNAs in many types of cancers were reported in different types of cancers, including breast, colon, gastric, lung, prostate and thyroid [20-24]. The peer-reviewed scientific literature on miRNAs is huge and indicated by ~15,943 PubMed hits as of March 2015 and, their role in cancer is very diverse both in terms of the disease and experimental approaches used by the investigators. Although the overwhelming majority of published papers focus on individual mRNA target, most miRNAs can exert their effects by targeting multiple mRNAs, some of which may reside in the same cellular pathway. Some studies have also shown that there were redundant with distinct sequences which can repress the same target mRNA [25]. Mouse models featuring miRNA overexpression or ablation have demonstrated causal links between miRNAs and cancer development [26,27].

### Bioinformatics methods to predict paradigms

The bioinformatics methods which are currently used to predict paradigms suggested that the interaction of miRNAs with their targets (candidate mRNAs) depends on the sequence, and evolutionary conservation [28,29]. Such methods identifies tens or hundreds of targets for each miRNA: however, the false positive rates were reported to be high [30]. Therefore, investigations examining the gain and loss of function of miRNAs are still needed to confirm the predictions. Evaluation of the association between a particular miRNA to a specific type of cancer is additionally complicated by the genetic diversity of tumors, and in cell lines derived from different tumors. A particular miRNA may have exhibited its oncogenic function in some types of cancers whereas the same miRNA was reported to act as a tumor suppressor in other cancers. Some such examples include: (i) miR-29, specifically miR-29a/-b/-c was reported as an oncogene in breast cancer while the same miRNA-29 acted as a tumor-suppressor gene in lung tumors [31,32]; (ii) loss of miR-23b conferred proliferative advantage and promoted bladder cancer cell migration and invasion [33] while knocking down the expression of same miRNA-23b in renal cell carcinoma (RCC) cell lines induced apoptosis and reduced invasive capabilities [34]. One possible explanation is that the same miRNA can participate in distinct pathways, having different effects on cell survival, growth and proliferation that are dependent on the cell type and pattern of gene expression. Furthermore, the potential for miRNA-mediated regulation of gene expression is enormous since ~60% of mRNAs are predicted to be under the control of miRNAs [35]. Hence, it is imperative to verify the phenotype and function of miRNA in appropriate animal and human cancer cell models.

### Abnormal expression of miRNA

There were several reports indicating widespread disruption of miRNA expression levels in numerous diseases, including cancer. Tumor tissues and cultured tumor cells often exhibit significantly reduced expression levels of mature miRNAs [36]. Different mechanisms for the aberrant expression of miRNA were documented. Three of them, viz., (i) genetic alterations and single nucleotide polymorphism (SNP), (ii) epigenetic silencing and (iii) defects in the miRNA biogenesis pathway, are discussed below.

(i) Genetic alterations and SNP: Complete mapping of human miRNA genes revealed that a great majority of the miRNAs were associated with fragile sites, cancer-specific translocation breakpoints, repetitive sequences and CpG islands [37]. However, some studies have indicated such association is not straight-forward and appears to be dependent on the specific type of cancer [38]. Furthermore, the existence of polymorphism in single nucleotides (SNPs) is widely known and, evidence has been presented suggesting the influence of SNPs on miRNA targets in cancer-related pathways [39]. A gain in function due to SNP may enhance its interaction with miRNA target and thus, enhance its regulatory function such as a tumor suppressor gene. In contrast, loss in function due to SNP may result in increased expression of miRNA, which then acts as an oncogene [40]. Additionally, SNPs in target sites of miRNAs may also result in the escape of degradation by miRNA [41]. All these observations suggested that SNPs may be one of the contributing factors in the regulation of biogenesis and functionality of miRNAs.
(ii) Epigenetic regulation of miRNA expression: Several research groups have investigated whether epigenetics, i.e., hyper- or hypo-methylation (an early event in carcinogenesis), play a role and influence the activity of miRNA genes [42-44] since the expression of miRNA genes, especially those located near CpG islands, tends to be affected more readily by methylation processes [42,43,45]. In scientific literature, there were several examples of DNA methylation processes influencing the activity of miRNAs. Some such examples were as follows. (i) The comparative analysis data in colon cancer cell line indicated that the expression of about 10% miRNAs tested were regulated by DNA methylation and that partial methylation reductions were not sufficient for the recovery of miRNA [46]. (ii) Screening investigations in colorectal cancers identified (a) epigenetic silencing of miR-34b and miR-34c due to hyper-methylation of neighboring CpG islands and (b) alteration in the methylation process affected miR-9 family genes [47]. (iii) Methylation of miR-9-1 was reportedly associated with lymph node metastasis in colorectal cancer cells (CRC) [48]. (iv) Significant and positive correlation between methylation of miR-200c/141 and invasive capacity of breast cancer cells [49]. (v) Methylation of miR-200c/141 is tightly associated with the invasive capacity of breast cancer cells [49]. (vi) In non-small cell lung cancer, promoter methylation was related with loss of miR-200c expression which in turn was associated with poor differentiation, lymph node metastasis and weaker E-cadherin expression [50]. In addition to DNA methylation, histone acetylation was also reported to be another epigenetic phenomenon in deregulated cancers. In breast cancer cells, histone deacetylase inhibition was shown to result in alteration in miRNA levels [51]. In bladder cancer cells, a combined treatment with 5-aza-2′-deoxycytidine (5-Aza-CdR) and histone deacetylase (HDAC) inhibitor 4-phenylbutyric acid (PBA) had a significant effect on multiple miRNAs among which miR-127 was most differentially expressed [45]. Specific induction/activation of miRNA-127 by 5-Aza-CdR and PBA suppressed the transcription of the zinc-finger repressor BCL6 gene and thus induced apoptosis in human cancer cells [45].
(iii) Defects in the miRNA pathway: In humans, the majority of miRNAs are encoded by introns of non-coding or coding transcripts. However, some miRNAs were reported to be encoded by exonic regions. The genes controlling miRNA are often clustered and transcribed as polycistronic messages or excised from mRNAs [52]. The precise locations of promoters for most miRNA genes are not yet mapped but, they can be inferred from collective analysis of CpG islands, RNA sequencing and chromatin immune-precipitation followed by ChIP-sequencing [53]. Numerous Pol-II associated transcription factors were reported to activate or repress several miRNA genes. The abundance of some miRNAs were also shown to be regulated at the RNA stability level [54]. Recently, Ser/Thr protein kinase/endoribonuclease IRE1α has been shown to be activated by endoplasmic reticulum stress and cleaved some selected pre-miRNAs, such as pre-miR-17, pre-miR-34a, pre-miR-96 and pre-miR125b, leading to translational reduction in the pro-apoptotic caspase 2 [55]. Previous reports have also indicated that Myc gene is responsible for up-regulating the oncogenic miR-17-92 cluster. The principal effect of Myc activation was the repressed expression of multiple miRNA [56,57] as well as down-regulation of several anti-proliferative, pro-apoptotic and tumor suppressor effects such as let-7, miR15a/16-1, miRNA-26a and miR-34 family members [58]. Similarly, activation of Ras gene was shown to result in repression of the miR-143/145 cluster in k-Ras mutant pancreatic cancers [59]. The p53 tumor suppressor gene was reported to regulate the expression of several miRNAs, such as miR34, miR-200. miR 15/16, etc. [60,61] and the miR34 family targets cyclin D and E2, CDK4, CDK6, Myc and BCl2 all of which play a major role in promoting cell proliferation, apoptosis and, these observations suggested the possibility that p53-induced miR-34 may negatively regulate cell growth [60,62].

### miRNA in cancer diagnosis and therapy

Microarray analysis of oligonucleotide miRNA is the most commonly used high-throughput technique for the assessment of the expression levels of hundreds of miRNA in a large number of cancer-specific cell types [63,64]. Studies using miRNA profiling have shown significantly different miRNA profiles in cancer cells compared with those in normal cells in the same tissue. Hierarchical clustering analyses also indicated that miRNA signature profiling enabled the tumor tissue samples to be grouped into a specific origin. Several genome-wide profiling studies have been performed on various types of cancers, such as breast, chronic lymphocytic leukemia, colon, lung, glioblastoma and thyroid papillary carcinoma, etc. [21,22,65-68]. Analysis of miRNAs in 76 breast cancer and 10 normal breast tissue samples had identified significantly dysregulated miR-125b, miR-145, miR-21 and miR-155: from such analysis, 15 such analyses could correctly predict whether the sample was normal or tumor breast tissue [22]. In a separate and similar investigation using breast cancer tissue, let-7d, miR-210 and miR-221 were found to be down-regulated in the ductal carcinoma *in situ* while they were up-regulated in the invasive transition [69].

### Non-invasive and inexpensive methods

Researchers are focusing on the examination of body fluids such as plasma, serum, urine and saliva to determine the circulating levels of miRNAs and to evaluate if they can be used as diagnostic, prognostic and predictive biomarkers in cancer. Such studies have attracted a great deal of attention because of minimally invasive processes to examine miRNA using qPCR. In the serum of prostate cancer patients, the expression levels of pre-selected oncogenic miR-26a, miR-195 and let-7i were shown to be up-regulated compared to those in individuals with benign prostate hyperplasia (BPH) [70]. Similarly, the prognostic value of increased expression levels of circulating miR-141 and miR-375 correlating with low-risk through high-risk and from localized to metastatic prostate cancer was documented [71,72]. The signature miRNAs, miR-28-3p, miR-30c, miR-92a, miR-140-5p, miR-451 and miR660 in the plasma were found to be deregulated 1–2 years prior to diagnosis of lung cancer and thus, indicated their use in prediction as well as diagnosis [73]. miR-27b, miR-158a, miR-326 signature or miR-200c in the serum of colon cancer patients were found to be useful to identify metastatic tumors [74,75]. The miR-125b and miR-155 levels in the serum of breast cancer patients were found to be useful for diagnosis, assessing chemotherapeutic response as well as in prognosis [76,77]. Until recently, miRNA analyses were performed using qRT-PCR and microarray-based approaches. NGS is now emerging as a cost-effective option while bioinformatics analyses are no longer a major problem for continued usage [78,79].

## Clinical trials

The increasing understanding of the molecular alterations underlying carcinogenesis and cancer had created opportunities to use miRNAs as diagnostic and prognostic indicators. Many signature miRNAs have been identified, and their use has been increasingly investigated in clinical trials in several countries including USA. For example: (i) circulating miRNA are used in breast cancer as biomarkers to examine therapeutic response (https://clinicaltrials.gov/ct2/show/NCT01722851); (ii) miR-10b is used in Glioma as biomarker to grade the tumor, survival and genotypic variation (https://clinicaltrials.gov/ct2/show/NCT01849952); (iii) miR-29 family (miR-29a/-b/-c) is used to investigate the role of Twist-1-mediated metastasis in Head and neck squamous cell carcinoma (https://clinicaltrials.gov/ct2/show/NCT01927354); (iv) circulating levels of miRNAs are evaluated as biomarker of response to treatment in Ovarian cancer (https://clinicaltrials.gov/ct2/show/NCT01391351); (v) multiple miRNAs are investigated to examine the response to chemotherapy in Non-small-cell lung cancer (https://clinicaltrials.gov/ct2/show/NCT00864266), etc.

## Conclusion

So far, there have been significant scientific research findings indicating the utility of miRNAs as biomarkers for prediction, diagnosis and prognosis. Evidence is also emerging suggesting that inhibition of oncogenic miRNAs or substitution of tumor suppressive miRNAs could be used to develop novel treatment strategies. The extensive information thus far available in the peer-reviewed scientific publications has been extremely useful to provide guidance for further investigations. Comprehensive, carefully designed, multi-centered, retrospective and prospective studies involving large cohorts in the same and independent laboratories/clinics comparing and validating the data within a similar type of cancer are warranted. Besides, investigations using minimally invasive methods to collect blood, saliva and urine are extremely important for the development of reliable and cost-effective miRNA-based technology for routine use in the clinics for early cancer diagnosis/detection and therapeutic assessment/prognosis.

## Abbreviations

miRNAs: microRNAs
RISC: RNA-induced silencing complex
UTR: The 3′ untranslated region
SNP: Single nucleotide polymorphism, 5-Aza-CdR, 5-aza-2′-deoxycytidine
